# DNA virus tropism in healthy dental pulp: an in-situ reservoir site for torque teno virus and parvovirus B19

**DOI:** 10.1080/20002297.2025.2499924

**Published:** 2025-05-01

**Authors:** O. I. Mielonen, S. Hänninen, J. Willberg, T. Salo, M. Mauramo

**Affiliations:** a Department of Oral and Maxillofacial Diseases, University of Helsinki, Helsinki, Finland; b Department of Pathology, University of Helsinki and Helsinki University Hospital, Helsinki, Finland; c Department of Pathology, Research Program in Systems Oncology, Research Programs Unit, Faculty of Medicine, University of Helsinki, Helsinki, Finland; d Department of Oral Pathology and Oral Radiology, University of Turku, Turku, Finland

**Keywords:** Virology, DNA viruses, odontology, human hard tissue, qPCR, Technovit® 9100, RNAscope in-situ hybridization

## Abstract

**Background:**

The presence of viruses in healthy teeth has not been extensively studied, although some viral traces have been detected in both healthy and diseased dental pulps in previous studies focusing primarily on a single species. The aim of this study is to clarify the persistence of DNA viruses in dental tissues and their impact on tissue composition.

**Materials and Methods:**

Here, the prevalence of persistent DNA viruses in intact third molars (*n* = 17) was assessed via quantitative PCR to detect human parvovirus B19 (B19V), torque teno virus (TTV) and nine human herpesviruses. Also, H&E-stained tissue sections of the samples were analyzed for any potential inflammatory process. RNAscope in-situ hybridization was performed for B19V, TTV and HHV7 subsequently.

**Results:**

Viral DNA of five different viruses was detected in 5 of the 17 samples (29.4%) including B19V (*n* = 2), TTV (*n* = 2), HHV7 (*n* = 2), HCMV (*n* = 1) or EBV (*n* = 1) in dental pulps with no signs of cytopathic effect, inflammatory cell accumulations or necrosis. RNAscope in-situ hybridization confirmed the presence of B19V and TTV in non-inflamed pulp tissue.

**Conclusions:**

These findings emphasized that even in the absence of a disease evaluated by histology, dental pulp can harbor DNA viruses and be an anatomical site of virus tropism, suggesting viral persistence rather than direct pathogenic activity.

## Introduction

Humans are continuously exposed to microbes, some of which persist even after the initial infection. There is a significant yet often overlooked component of the microbiome: the large population of DNA viruses [1] that can persist for a lifetime in human tissues, forming a human virome. Every individual and tissue type possess a unique virome, which is rarely present in peripheral blood [2], although previous virus screening studies have frequently focused secretions and fluids, e.g. whole blood, urine and saliva instead of solid organ samples. Compared to solid tissues these can be considered more accessible specimen types but as a drawback they capture only fractions of total tissue virus prevalence [3]. Thus, our understanding of viral tissue tropism, latency processes and the health effects of viral persistence is still quite limited.

Due to the direct anatomical connection to the environment the oral cavity serves as a route of transmission for many viruses, and some of these may also cause infections in the oral cavity mucosa making it a compelling target for studying viral etiology. While the role of viruses as pathogens in oral inflammation is gaining increased research attention [4], it remains uncertain whether viruses are responsible for pathogen-associated diseases affecting dental and surrounding tissues, such as periodontal [5] or endodontic infections [6] For instance, the association between herpes viruses and periodontal infections is better established than with endodontic infections, advocating more research, such as longitudinal studies, to address potential causal aspect [7]. Consequently, understanding viral prevalence and tissue preference in the oral cavity free of disease is crucial for comprehending the broader impact of viruses on oral infections.

Histologically confirmed normal dental pulp tissue has not been yet subjected to systematic DNA virus screening and even though several healthy oral sites are acknowledged lytic/latent targets of viruses [4], it is unclear how prevalent common persistent DNA viruses are in dental tissues and whether they are bystanders in a rather passive state without affecting tissue composition or somehow associated with tissue pathology.

In this study, we analyzed a panel of 11 DNA viruses by qPCR from the pulps of intact third molars collected during routine tooth extraction (*n* = 17) to compare the histological findings. Moreover, the specific locations where DNA viruses might reside within dental tissue are still unknown. Therefore, another goal of this study was to deepen our understanding of this phenomenon through in-situ analysis.

## Materials and methods

### Ethical statement

The study protocol was approved by the Ethical Committee of the Northern Ostrobothnia Hospital District (23–2003). The study has been conducted according to the principles expressed in the Declaration of Helsinki, 1975. Individual consents were not requested due to the study’s emphasis on maintaining anonymity. Personal or clinical registry data was not processed.

### Sample collection and preparation

Macroscopically intact third molars (*n* = 17) were extracted due to impaction, crowding or preventive measure and collected during routine clinical dental care in 2007–2017 at Finnish Student Health Service (Oulu, Finland). The teeth were immediately stored in ethanol and preserved at  −20°C for longer periods.

The surface of frozen teeth was first mechanically cleaned with a mix of ethanol 75%–85%, detergent soap and water. For in-situ analysis, horizontal cross-section from each tooth was cut in the transverse plane at the cemento-enamel junction using a diamond-cutting disc and stored in  −80°C. Following this, residual pulp-dentin complex was grinded by drilling for qPCR analysis.

Sample handling was performed in enclosed laminar hood with sample-specific cover foils and gloves. Surfaces and equipment (drill, drill bits/discs, forceps) were cleaned with hypochlorite (2,5 g per 100 g) and 70% ethanol in the beginning and between individual sample handlings.

### DNA extractions and virus detection of pulp-dentin complex

Extraction of DNA for qPCR analysis was performed by QIAamp® DNA Investigator Kit (Qiagen) according to manufacturer’s protocol for bones and teeth. The DNA was eluted to 100 μl of ATE buffer and stored in  −20°C until the viral assays were conducted.

The quantifications of human parvovirus B19 (B19V), torque teno virus (TTV) and nine human herpesviruses (herpes simplex virus 1 (HSV1) and 2 (HSV2), varicella zoster virus (VZV), Epstein–Barr virus (EBV), human cytomegalovirus (HCMV), HHV6A, HHV6B, HHV7 and HHV8) were performed as described earlier [8–10] with AriaMx Real-Time PCR System or Stratagene M×3005P qPCR System, both by Agilent. Also, the human RNase P gene was amplified to demonstrate the cell count in all samples and to normalize virus copy numbers (viral copies/million human cells), as described [8]. All qPCR reactions were run in duplicates.

PCR-grade water was used as a negative control in all steps, starting from the beginning of DNA extraction. In order to avoid contamination, DNA extraction and each step of qPCR (reagents, samples, plasmids) were performed in laminar hoods of separate rooms. Throughout the process, filtered tips and single-use PCR consumables were used.

Of sample no. 9, a hard tissue section was prepared using Technovit®9100 embedding method described earlier [11] with some modifications to examine RNA distribution in a cross-section of the tooth. The sample was fixed in formalin for 16 h and the infiltration was performed for 1 week in  −20°C. Molding was performed in  −20°C for 1 week and after that the samples were transferred for 1 night to  +4°C. This method preserved the anatomical structure, including both the pulp and the surrounding dentin and enamel.

### Sample fixation, preparation of the tissue sections and histopathological analysis

All samples from the rest of the cohort (*n* = 16) were fixed overnight in 10 % neutral buffered formalin at  +4°C. The pulp was separated from dentin, dehydrated in a series of graded ethanol + xylene, paraffin embedded, and cut by microtome as 5 μm sections and placed on SuperFrost Plus slides. Sections were then stained with hematoxylin and eosin (H&E). H&E-stained tissue sections were analyzed for any potential inflammatory process by a blinded pathologist (MM), and scored to 0, when most fields (>7/10 hPF) had no inflammatory cells; 1, when most fields (>7) had < 35% of the area filled with inflammatory cells; and 2, when most fields (>7) had more than 35% filled with inflammatory cells [12]. Additionally, collagen degradation, necrosis, vascular changes and pulp tissue organization were assessed [13].

### RNAscope in-situ hybridization

RNAscope in-situ hybridization (RISH) was performed for hard tissue section no. 9 and samples representing the most prevalent viruses (B19V, TTV, HHV7) using the RNAscope 2.5 hD Reagent Kit – RED (Advanced Cell Diagnostic, CA) according to the manufacturer’s protocol. Protease treatment and target retrieval were performed for 15 min. Both the human PPIB mRNA probe and the bacterial DapB probe served as positive/negative technical controls.

In the hard tissue section of no. 9 only PPIB and DapB probe signals were amplified as proof of the concept here. The slide was processed without a baking step. Also, deparaffinization was replaced with deacrylation (2 × 10 min in xylene, 1 h and 45 min in methoxyethyl acetate, 2 × 2 min in pure acetone and 1 min in aqua).

To detect the virus nucleic acids, V-B19V-NS probe targeting both virus DNA and messenger RNA (mRNA) was amplified for *NS* gene of B19V [14]. V-HHV7-U4 probe targeting mRNA for HHV7 *U4* gene, and V-TTV-2005-ORF1-O1-sense targeting antisense strand for TTV *ORF1* gene, were amplified respectively. Slides were scanned at x40 magnification and extended depth of focus by 3D HISTECH Pannoramic 250 Flash III scanner.

## Results

### Prevalence of DNA viruses in pulp-dentin complex

In this study, 11 DNA viruses (B19V, TTV, HSV1, HSV2, VZV, EBV, HCMV, HHV6A, HHV6B, HHV7, HHV8) were analyzed by qPCR from the pulp-dentin complex of macroscopically intact third molars (*n* = 17). Overall, five different viruses were detected in five samples out of 17 (29.4%) comprising of B19V (*n* = 2), TTV (*n* = 2), HHV7 (*n* = 2), HCMV (*n* = 1), and EBV (*n* = 1) (Table 1). Most of the positive samples presented one virus finding (*n* = 3). Also, a double positive (TTV, EBV) and a triple positive (HHV7, HCMV, TTV) co-occurrence was detected. While TTV (*n* = 2), EBV (*n* = 1), and HCMV (*n* = 1) occurred as co-findings, B19V (*n* = 2) was detected with no other viruses present in the same samples.

**Table 1.** Results of positive virus findings in pulp-dentin complex and copy numbers per million cells with respective sample numbers.

	HHV7	HCMV	TTV	B19V	EBV
1.				2140	
4.	1130				
10.	231	940	19500		
11.				2120	
12.			307		198

B19V = parvovirus B19, TTV = torque teno virus, EBV = Epstein-Barr virus, HCMV = human cytomegalovirus, HHV7 = human herpesvirus 7.

The RNase *P* values of frozen pulp-dentin samples ranged between 2 020–132 200 (median 14 060) copies/μl of DNA extract translating to 1010–66 100 (median 7030) cells/μl. The median for virus copies per million cells was 1 035 (range 198–19 500). The highest copy number was detected for TTV and lowest for EBV (Table 1).

### RNAscope in-situ hybridization and histology

Only PPIB and DapB probe signals were amplified in the hard tissue section of no. 9 by RISH. A weak positive signal acquired with PPIB probe concentrating only in pulp tissue was observed here. However, PPIB signal was not detected at the predentin–dentin interface, and thus only pulp tissue from the rest of the cohort was collected for H&E analysis and downstream assays to improve RISH sensitivity. Negative control DapB remained negative in the hard tissue section of no. 9.

Based on H&E analysis, the rest of the cohort (*n* = 16) displayed normal pulp microscopic architecture with no signs of cytopathic effect, inflammatory cell accumulations (grade = 0) or necrosis.

In virus RISH staining, 10 dots observed with 40× magnification were considered positive. This criterion was met with B19V (NS) in the sample no. 1 and TTV (ORF1) in the sample no. 10 also with the highest virus copy numbers in the cohort measured by qPCR (B19V 2140 and TTV 19 500 copies per million cells, respectively). However, HHV-7 (U4) showed no signal here.

Overall, the virus positivity in these samples was distributed on focal zones with high cell density, while the positive control probe PPIB exhibited scattered signal throughout the connective tissue matrix in every sample tested here. Negative control DapB remained negative in all tested samples.

**Figure 1.**
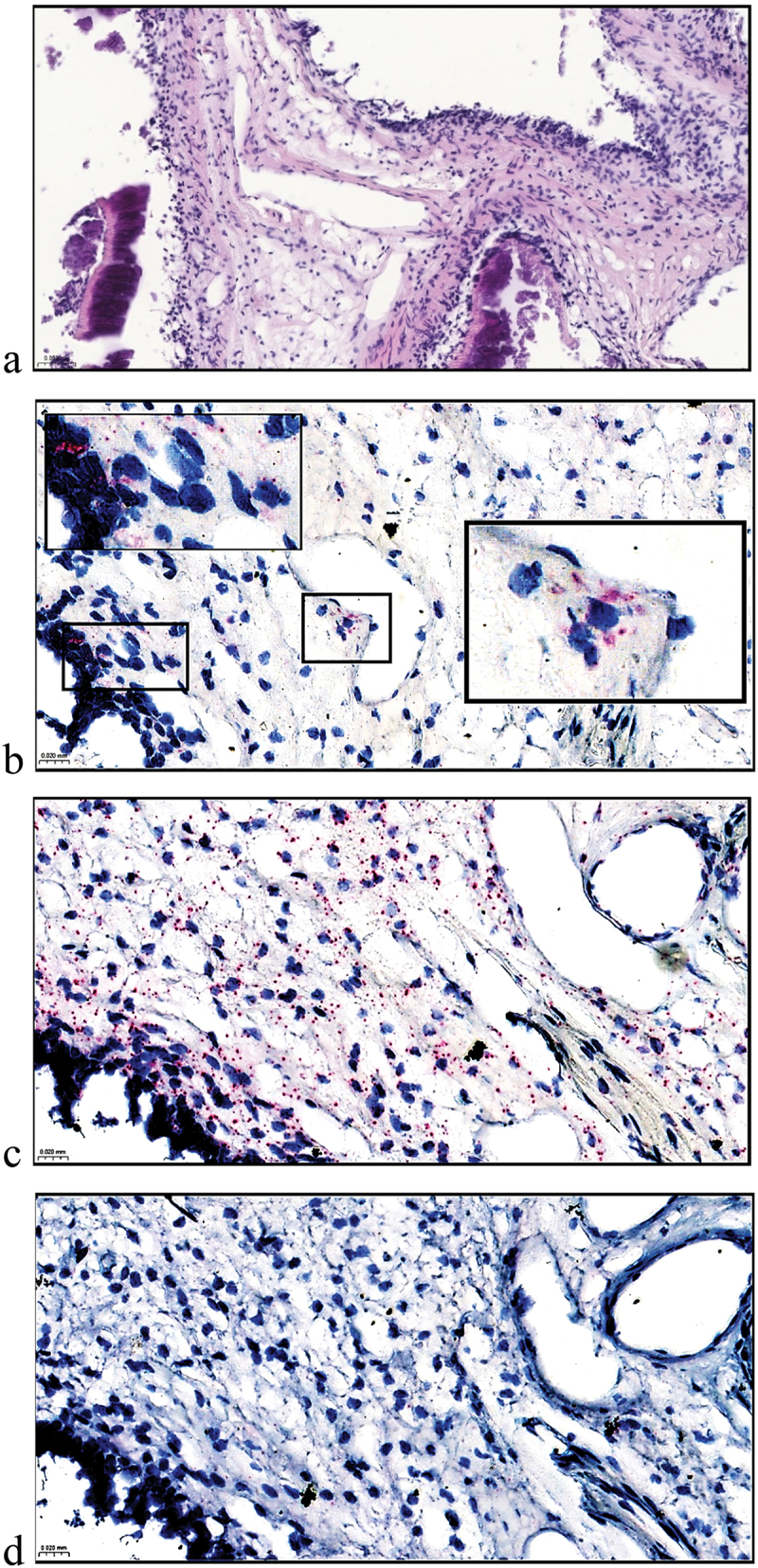
Histology and detection of B19V NS gene/transcript mRNA by RNAscope 2.5 hD assay in pulp no. 1. H&E staining demonstrating some fragmented dentin and pulp tissue with capillaries and fibroblasts within connective tissue surrounded by odontoblastic layer (a). Representative positive virus probe signals are indicated as higher magnifications of the areas (b). Positive and negative technical controls of human PPIB probe (c) and bacterial DapB probe (d) were included. Images captured with SlideViewer; a with 20× magnification. b-d with 40× magnification. Scale bars in the lower left corner; 50 μm (a) and 20 μm (b–d).

**Figure 2.**
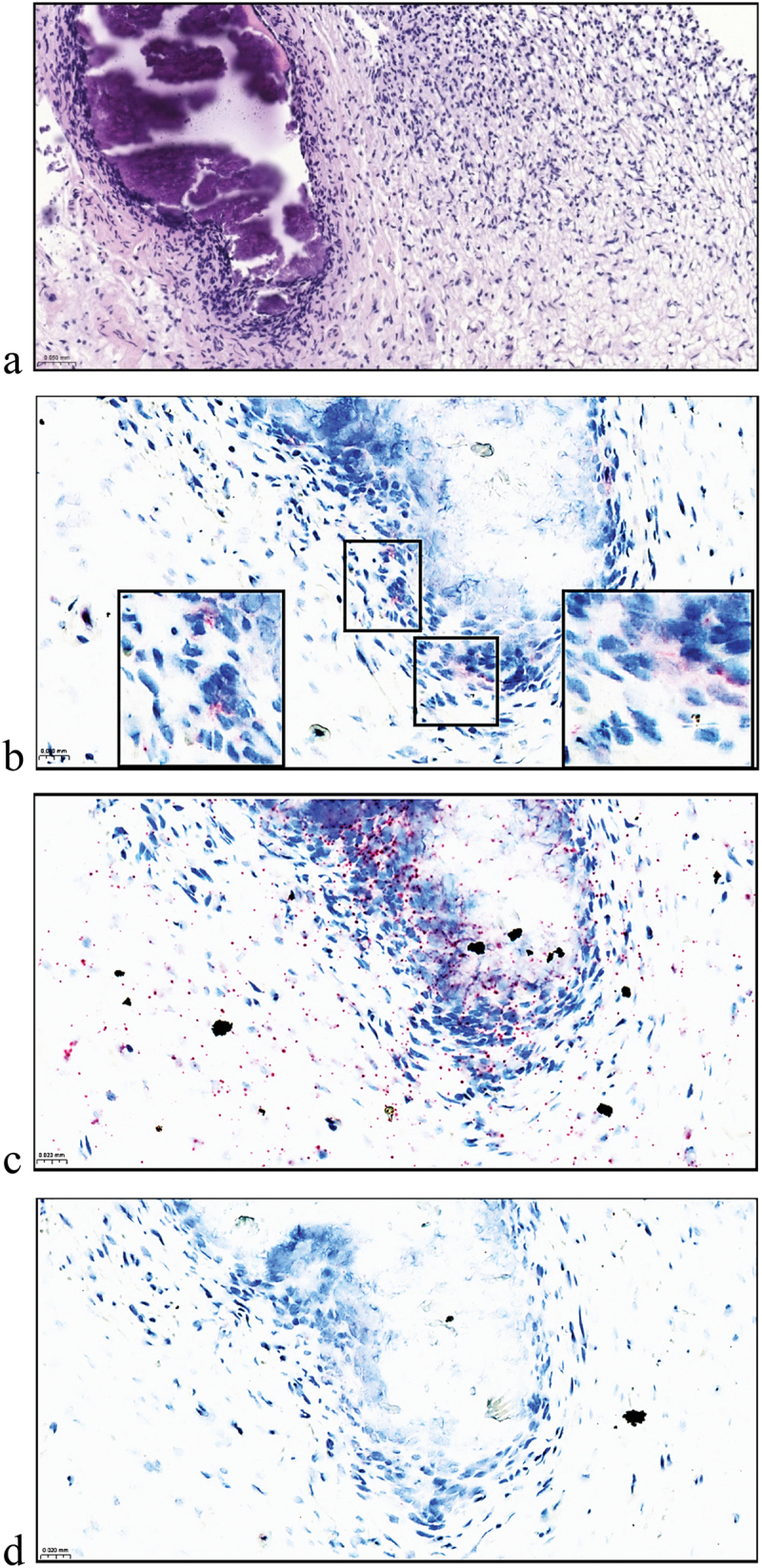
Histology and detection of TTV ORF1 gene by RNAscope 2.5 hD assay in pulp no. 10. H&E staining with focal cluster of fragmented dentine in the middle of a normal pulp tissue (a). Representative positive virus probe signals are indicated as higher magnifications of the areas (b). Positive and negative technical controls of human PPIB probe (c) and bacterial DapB probe (d) were included. Images captured with SlideViewer; a with 20× magnification and b-d with 40× magnification. Scale bars in the lower left corner; 50 μm (a) and 20 μm (b-d).

B19V positive dots were detected on cell-rich zone near odontoblastic layer and also in cells on connective tissue near vascular endothelial lining (Figure 1). Similarly, TTV showed a positive signal within a cell-rich zone scattered along a focal section of dentin surrounded by a normal pulp architecture (Figure 2).

## Discussion

In this study we examined the presence of DNA viruses in pulp-dentin complexes by qPCR from frozen intact third molars free of injury, decay and histopathological signs of inflammation. As a result, DNA of HHV7, HCMV, TTV, B19V, or EBV were detected in 5/17 samples. Subsequently, we performed RISH to visualize the location of the nucleic acids of the three most prevalent viruses comprising B19V (*n* = 2), TTV (*n* = 2), HHV7 (*n* = 2).

In pulp tissue, little is known about the DNA virus-associated histological tissue landscape. Regarding RNA viruses, human immunodeficiency virus has been detected within fibroblasts of the non-inflamed pulp by in-situ hybridization method [15]. Hepatitis C seropositivity has been shown to change normal pulp tissue architecture, however without a direct detection of the virus from tissue [16].

Also, some earlier research has been conducted to determine the presence of DNA viruses in pulp and clinical periapical inflammation. EBV and HCMV have been the most associated with endodontic pathology, but the etiological role remains uncertain [17]. Interestingly, it has been suggested that herpesviruses and bacteria are synergistic in the pathogenesis of apical periodontitis e.g. by EBV-induced bone resorption through increased production of reactive oxygen species and bone resorption regulators [18]. In this study, the viruses detected within tissue did not appear to cause any viral cytopathic effect or inflammation. This might be relevant to consider before assessing any direct causal links between viruses and pulp/periapical inflammation, although this topic and especially more complex virus–host interactions are beyond the scope of the present study and require further follow-up research.

As previously noted, pulp has been identified as a source of viruses in earlier studies, and we observed some consistency also here, particularly in the prevalence of EBV and HCMV [19–23]. Additionally, *Anelloviridae* DNA, the family to which TTV belongs, has been isolated from an ancient pulp sample [24]. To our knowledge, there have been no previous reports of B19V and HHV7 in dental pulp. However, other viruses such as HHV8 [23], HHV6 [25], HSV1 [21–23] and VZV [26], which have been documented in earlier research, were not detected in our study. The small cohort size here might explain this variation. Furthermore, the low virus DNA quantities observed (≤19,500 per million cells) suggest a persistent rather than an acute infection, which typically makes detection more challenging [27].

RISH is a sensitive technique for visualizing specific nucleic acids within their original tissue context, with extravascular signals indicating that the findings are specific to the tissue and not solely derived from circulation. We examined the endogenous RNA distribution in dental tissue from hard tissue sections embedded in Technovit® 9100 using RNAscope in-situ hybridization – a combination of methods not previously described. PPIB RNA transcripts were primarily found in pulp tissue, leading us to focus on pulp for virus detection. This result was expected, as odontoblastic processes in dentine tubules lack nuclear DNA, though mitochondrial DNA is present [28]. Nevertheless, further research is warranted to optimize detection sensitivity and adjust background staining for improved signal with RISH in hard tissue sections.

The present study established first evidence of TTV and B19V tissue distribution in dental pulp. Common diseases caused by B19V include childhood rash erythema infectiosum (fifth disease). In contrast, TTV infection does not have any specific clinical symptoms, although higher copy levels are often associated with conditions involving inflammation [29]. B19V has a seroprevalence of 90% among older adults [30], while TTV is even more widespread, with a global prevalence of up to 95% [31]. Both pathogens persist after the initial infection and thus can be found in healthy individuals as well. Erythroid lineage cells, endothelial cells [32] and B lymphocytes [33] are the acknowledged sites of persistence for B19V. Dental pulp seems to lack B lymphocytes [34], but our discovery of B19V near endothelium aligns with the current understanding of its host site.

The primary target organ for TTV replication is still ambiguous, yet several sites including liver, bone marrow, lung, lymphoid tissue, blood mononuclear cells and granulocytes have been identified, suggesting a wide host cell tropism [35]. Due to high DNA levels of TTV in saliva, the oropharyngeal region has been investigated as a potential target for infection [4]. Elevated TTV levels have been detected in potential premalignant lesions of oral carcinoma [36] and in tooth supporting tissues of patients with periodontitis [37]. Considering oral host cell, TTV has been detected in oral epithelial cell cytoplasm by in-situ method [38] without a specific disease association. This is also the case in this study regarding dental pulp, which could suggest a low copy TTV presence being a part of the tissue virome rather than a trigger for inflammation in this context.

This study also has its limitations. First, the small cohort size may impact the generalizability of the findings. Second, the TTV RISH probe was targeted only at DNA, while the probe for B19V was not selective between mRNA and DNA [14]. Moreover, although we employed an mRNA-specific probe for HHV7, it yielded no signal due to low transcription or absence of it. The detection of viral DNA by qPCR or RISH alone does not confirm viral activity, highlighting the need for further investigations, such as mRNA studies, to accurately conclude this. Third, the strictly anonymized data of the study posed some challenges, such as the lack of clinical information of accompanying medical conditions, especially in oral cavity. Including that would be both insightful and valuable, as it could uncover potential connections between health events and the viruses found in the pulp.

Despite these limitations, the study’s strength lies in the use of well-preserved clinical specimens, where DNA virus in-situ distribution has not been thoroughly explored before. According to these findings, non-inflamed dental pulp can harbor virus DNA without morphological changes to the tissue. This result indicates viral persistence rather than direct pathogenic effect and thus provides information on viral prevalence and tissue preference in the pulp, which is crucial for comprehending the broader impact of viruses on infections in this anatomical site, where pathogen invasion normally leads to inflammation and necrosis. Thus, by screening for a diverse group of viruses in this patient material, we have gained valuable insights into the association of virome in normal pulp tissue free from inflammation or disease, establishing a baseline for future research.
